# Co-creating strategies to promote uptake of HIV self-testing among young adults in Mecklenburg county, North Carolina: a protocol for a pilot implementation study

**DOI:** 10.3389/frhs.2025.1536236

**Published:** 2025-03-28

**Authors:** Ucheoma Nwaozuru, Lindsay Miller, Laura H. Gunn, Sebastian Marin-Cespedes, Margaret Hanff, Patrick Robinson, Michael Dulin, Meghana Muralidhar, Prashant Jha, Goodness C. Mirikwe, Donaldson F. Conserve, Chelsea Gulden, Bernard A. Davis, Kristie Foley, Joseph Tucker, Meagan Zarwell

**Affiliations:** ^1^ Department of Implementation Science, Division of Public Health Sciences, Wake Forest University School of Medicine, Winston-Salem, NC, United States; ^2^ Department of Epidemiology and Community Health, College of Health and Human Services, University of North Carolina at Charlotte, Charlotte, NC, United States; ^3^ School of Data Science, University of North Carolina at Charlotte, Charlotte, NC, United States; ^4^ School of Public Health, Faculty of Medicine, Imperial College, London, United Kingdom; ^5^ Department of Health Policy and Management and the Academy for Population Health Innovation, College of Health and Human Services, University of North Carolina at Charlotte, Charlotte, NC, United States; ^6^ College of Health and Human Services, University of North Carolina at Charlotte, Charlotte, NC, United States; ^7^ Musculoskeletal Institute, Atrium Health Carolinas Medical Center, Charlotte, NC, United States; ^8^ Departments of Biology and Chemistry, Wake Forest University, Winston-Salem, NC, United States; ^9^ Department of Prevention and Community Health, Milken Institute School of Public Health, George Washington University, Washington, DC, United States; ^10^ RAIN Inc., Charlotte, NC, United States; ^11^ RAO Community Health, Charlotte, NC, United States; ^12^ Department of Medicine, Division of Infectious Diseases, University of North Carolina at Chapel-Hill School of Medicine, Chapel-Hill, NC, United States; ^13^ Clinical Research Department, Faculty of Infectious and Tropical Diseases, London School of Hygiene and Tropical Medicine, London, United Kingdom; ^14^ Violence Prevention Center, University of North Carolina at Charlotte, Charlotte, NC, United States

**Keywords:** crowdsourcing, community-based participatory approaches, young adults, HIV self-testing, North Carolina

## Abstract

**Background:**

HIV testing is the gateway to entering HIV care and prevention services. However, HIV testing rates remain low among young adults (18–29 years old) in Mecklenburg County, North Carolina (NC), an ending the HIV epidemic (EHE) priority jurisdiction. We aim to utilize community-engaged and participatory approaches to co-create implementation strategies to promote the reach and uptake of HIV self-testing (HIVST) among young adults in the region. This study protocol outlines the phases of the project and the proposed outcomes.

**Methods:**

The Community-engaged Approaches to Expand HIV Self-Testing among Young Adults in Mecklenburg County, North Carolina (CATEST) project will be conducted in three phases, guided by the Consolidated Framework for Implementation Research (CFIR), Community-based Participatory Research (CBPR), and Reach, Effectiveness, Adoption, Implementation, and Maintenance (RE-AIM) frameworks. The formative phase of the study, guided by CFIR, will focus on understanding the barriers, facilitators, and opportunities for implementing HIVST among young adults in Mecklenburg County, North Carolina. The second phase, guided by CBPR, will utilize participatory approaches such as crowdsourcing open calls and charrettes to co-create implementation strategies for HIVST. Then, the final pilot implementation phase, guided by CFIR and RE-AIM, will use mixed methods to evaluate the success of the co-created HIVST implementation strategies using a pre-post design. Participants in the study will complete a baseline survey and a follow-up survey immediately following intervention completion. In addition, a purposive sample of participants and representatives at the participating community organization will complete qualitative exit interviews within 1 month of intervention completion.

**Discussion:**

This study protocol outlines the co-creation of implementation strategies, tests their feasibility, and explores preliminary effectiveness in promoting HIVST uptake among young adults in Mecklenburg County, NC. The study will yield insights on the feasibility of leveraging the capabilities of community and youth innovation to promote young adults-centered implementation strategies to advance the reach and adoption of HIVST among young adults.

**Registration:**

Registered on Open Science Forum-DOI 10.17605/OSF.IO/2BZWV.

## Background

In the U.S., HIV incidence among young adults who are 20–29 years old is disproportionately high, with over a quarter of all new infections occurring among this group in 2019 (1). Despite the high HIV burden, HIV testing rates among young adults remain low (2). Knowledge of HIV status is key to early access to HIV treatment and prevention (3). An estimated 15% of people living with HIV in the U.S. are unaware of their infection (4), and young adults accounted for 21% of new diagnoses in 2019 and are the group least likely to be aware of their HIV status secondary to low rates of testing (4). Syndemic theory suggests that HIV risks are not a singular phenomenon but are influenced by social factors such as intersectional stigma, mental health, income/poverty, substance use, and other social conditions (5). Current programs to promote HIV testing among young adults have had limited impact, partly due to limited young adults' engagement in developing HIV testing strategies and addressing syndemic factors (6–8).

Increasing HIV testing among young adults is critical to advancing HIV prevention efforts and for the U.S. to meet the Ending the HIV Epidemic (EHE) goal of a 90% reduction in HIV incidence by 2030 (9, 10). EHE aims to prevent new HIV infections by focusing on high-risk communities, including Mecklenburg County, which has the highest rate of new diagnoses in North Carolina (NC) (11). Young adults in Mecklenburg County are at increased risk for HIV acquisition and have one of the lowest HIV testing rates (11, 12). HIV self-testing (HIVST), which allows individuals to self-administer HIV tests in private and interpret their results (13, 14) has shown promise in mitigating barriers to testing by decentralizing HIV testing and decreasing test-related stigma (14–16). Recent studies demonstrate challenges in reach, uptake, and linkage to post-HIVST preventive/care services [e.g., antiretroviral therapy (ART) for people with HIV (PLHIV) and pre-exposure prophylaxis (PrEP) for those without HIV], which limits its impact among populations such as young adults who will most benefit from this innovation (16). Thus, to maximize the impact of HIVST, novel strategies that center the voices of young adults in the promotion and delivery of HIVST are needed.

We propose an innovative approach to expand the reach and uptake of HIVST among young adults in Mecklenburg County, NC, informed by principles of Community-Based Participatory Research (CBPR) (17, 18). This study, Community-engaged Approaches To Expand HIV Self-Testing (CATEST), will involve crowdsourcing open calls, a Community-based Participatory Research (CBPR) approach to involve the members of underrepresented and historically marginalized populations and community partners in the development of young adult-informed HIVST strategies solutions that take syndemic factors into account. Crowdsourcing is an approach that engages community members to generate solutions to a problem and then implements selected community-engaged solutions (8, 19, 20). Crowdsourcing has been effectively used to improve HIV testing and condom use (21). However, to our knowledge, no studies have co-created and piloted HIVST implementation strategies for young adults in the Southern U.S.

The CATEST study directly responds to the urgent need to engage young adults in HIV prevention services in Mecklenburg County, which has the highest rate of new diagnoses in NC (22, 23). This manuscript outlines the protocol for the study phases and proposed outcomes.

### Guiding frameworks

The study is guided by the principles of CBPR (17, 18). CBPR seeks to address disparities in knowledge production by involving community partners as the experts of their own experiences, local contexts, and ideas for problem-solving (17, 18). To ensure broad community representation for this study, we will work with established young adult-serving organizations, HIV organizations, and community members in Mecklenburg County, NC, to guide recruitment, facilitate relationships, and recommend community-driven approaches from the crowdsourced strategies. In addition, during the preparation of the study proposal, we incorporated feedback from representatives of young adult-serving community organizations and a representative from the Mecklenburg County Health Department. CBPR will inform the crowdsourcing open calls and charrettes.

In addition, two implementation frameworks - Consolidated Framework for Implementation Research (CFIR) (24) and Reach, Effectiveness, Adoption, Implementation, and Maintenance (RE-AIM) (25, 26) - will guide the implementation evaluations. CFIR (24) constructs include (a) intervention characteristics, (b) outer setting, (c) inner setting, (d) characteristics of individuals receiving or delivering the intervention, and (e) implementation process. These constructs are widely used to evaluate pre-, post-, and ongoing implementation processes (27, 28). CFIR will be used during the study's pre-implementation phase to assess barriers and facilitators to implementing HIVST among young adults. Findings from the pre-implementation phase will inform a contextually tailored crowdsourcing open call to generate HIVST implementation strategies. In addition, CFIR will guide the exit interview in the post-implementation phase to understand barriers and facilitators to implementing the strategies. The RE-AIM framework will guide the implementation and post-implementation phases, evaluating the RE-AIM domains of these strategies (25, 26). We will include all five domains: *Reach* (number of young adults who enroll in the intervention and number of HIVST distributed), *Effectiveness* (impact of the intervention on clinical outcomes testing), *Adoption* (number of participants recruited), *Implementation* (extent of intervention delivery), and *Maintenance* (sustainability of outcomes) (25, 29).

**Figure 1:**
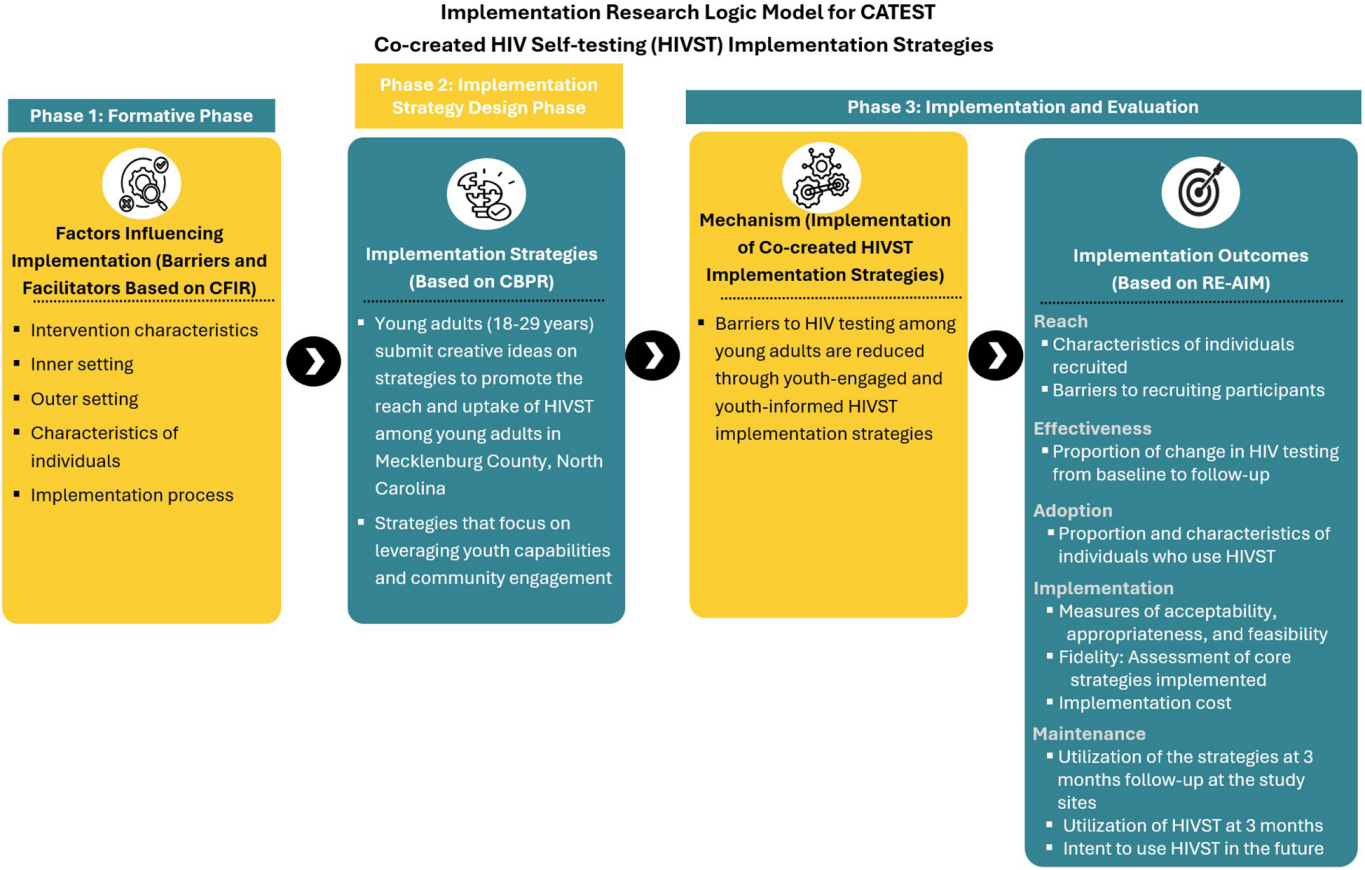
Conceptual model for the CATEST study. The model draws from the Consolidated Framework for Implementation Research (CFIR) (24), Community-Based Participatory Research Program (CBPR) (17, 18), and Reach, Effectiveness, Adoption, Implementation, and Maintenance (RE-AIM) (25, 26) frameworks.

The implementation logic model for this study (Figure 1) highlights the formative/pre-implementation phase of the study guided by CFIR (24), participatory and innovative strategies for co-creating and selecting implementation strategies using crowdsourcing open calls and charrettes guided by CBPR (17, 18), assessment of multi-level determinants during implementation guided by CFIR (24), and assessment of implementation outcomes guided by RE-AIM (25, 26).

## Methods

### Study setting

All research activities will take place in Mecklenburg County, NC (11). In 2022, Mecklenburg County had an estimated population of 1.12 million, with a median age of 35.4, and approximately 22% of the population between the ages of 18 and 29 (30). Mecklenburg County has the highest rate of new HIV diagnoses in the state and is one of the 48 recognized priority county jurisdictions for the federal EHE plan (23). New HIV cases are highest among males (82%) and black persons (42%), with individuals aged 18–29 years contributing an estimated 24%–47% of new HIV infections (31).

### Community advisory group (CAG)

We will conduct the study in collaboration with diverse communities of interest in Mecklenburg County, including young adults and community partners from young adult-serving HIV organizations. We have convened an initial community advisory group (CAG) comprised of ten young adults ages 18–29 years to guide the research activities, including providing feedback on recruitment materials, participant recruitment, and overall research activities. Additional CAG members will be recruited with the help of the existing CAG members serving as young adults and recruitment champions. Our current CAG members helped review and develop this study protocol. CAG members will be compensated US$50 for each meeting and will also be provided with meals for in-person meetings.

### Study design and overview of study aims

The study consists of three phases corresponding to its specific aims. The first phase is the formative phase. This initial phase involves in-depth interviews with young adults and other communities of interest, including representatives from organizations providing HIV services to young adults. The goal is to understand the barriers, facilitators, and opportunities for implementing HIVST among young adults in Mecklenburg County, NC. The second phase is the implementation strategy design phase. In this phase, we will utilize participatory approaches such as crowdsourcing open calls and charrettes to co-design implementation strategies for HIVST. These methods will engage communities in the design process to ensure the strategies are relevant and effective. This phase is informed by crowdsourcing open call activities, such as the 4 Youth By Youth (4YBY) project in Nigeria (31, 32) and other U.S.-based crowdsourcing efforts led by some members of the research team (8, 33, 34), which have demonstrated the value of leveraging the wisdom of the crowd to develop community-relevant and -driven interventions. The third phase will consist of implementing and evaluating the co-created implementation strategies for HIVST using a pre-post design.

#### Phase 1: formative phase - identify barriers and facilitators to implementing HIVST interventions among young adults in Mecklenburg county

##### Participants and recruitment

Participants include key community partners and young adults, selected through a purposive sample of staff and clients from local organizations that provide HIV testing and other HIV prevention and care services to young adults. This includes representatives from HIV service organizations, youth organizations, community health clinics, the local health department, and young adults in Mecklenburg County. We estimated 30 participants based on the prior experience of the research team (35, 36).

##### Procedure

We have completed 18 in-depth interviews thus far. Recruited individuals participated in semi-structured interviews in person or virtually, depending on their preference. Each interview lasted approximately 30–45 min. The interview guide was adapted for different groups of interviewees to adjust for relevant differences in roles and organizations. Interviews explore barriers and facilitators to implementing HIVST services and recommendations to encourage uptake among young adults. In addition, the interviews explore syndemic factors that influence the uptake of HIV testing among young adults. The interview is based on the CFIR framework (24) questions for partnering sites ask about (1) Intervention characteristics (relative advantage, complexity); (2) Outer setting (young adults' needs and resources, external policy & incentives); (3) Inner setting (relative priority, compatibility, implementation climate, networks & communications); (4) Characteristics of individuals (perceived knowledge and beliefs, self-efficacy); and (5) Implementation process factors (planning, reflecting, and evaluating). Probes and other prompts are used to explore emerging new lines of inquiry. Interviews capture participants' preferences, circumstances, perceptions, and young adult needs that shape HIVST uptake to inform future implementation strategies. Interviews were recorded with participant permission; audio recordings were transcribed, and transcripts were quality-controlled prior to analysis.

##### Analysis

Interview audio recordings were transcribed using a secure transcription service (Rev.com), entered into NVivo version 15 for coding, and organized for subsequent qualitative data analysis. We will use the framework approach for qualitative data analysis (37), a five-step process that involves: (a) Immersion in details of multiple data sources, gaining a general understanding of content and documenting initial impressions; (b) Developing a theoretical framework - to identify emergent themes in data sources, guided by existing theories (37). Themes will be refined and compared; (c) Indexing - further immersion in the data to refine themes and sub-themes; (d) Summarizing data using the CFIR analytical framework; and (e) Data synthesis and interpretation to compare themes and sub-themes against original transcripts, memos, notes, and audio recordings to ensure appropriate context (37). Two research team members will independently code data to improve its reliability and validity and enhance scientific rigor. Inter-rater reliability will be determined based on a subset of the data (i.e., 2–3 interview transcripts), and consensus meetings will be repeated until satisfactory rater agreement (80% of coded data) is achieved. This iterative and comparative process will continue throughout data collection until data saturation is reached. Established procedures to enhance the credibility of our analysis will be used (38), including analyses of codes that do not fit our coding scheme and the development of a decision audit trail. Results will inform the development of prompts for the crowdsourcing open call and be summarized for discussion during the charrettes process in Phase 2 of the study.

##### Expected outcome

Phase 1 of the study will generate insights into opportunities, barriers, and facilitators of existing strategies for implementing HIVST interventions. This will inform the crowdsourcing open call in Aim 2, which will focus on identifying strategies to mitigate salient barriers while leveraging key facilitators and opportunities identified.

#### Phase 2: implementation strategy design phase - apply participatory approaches (crowdsourcing open calls and charrettes) to develop HIVST implementation strategies for young adults in Mecklenburg county shows the steps for phase 2 of the study)

##### Participants and recruitment - crowdsourcing open call [phase 2.1]

**Figure 2:**
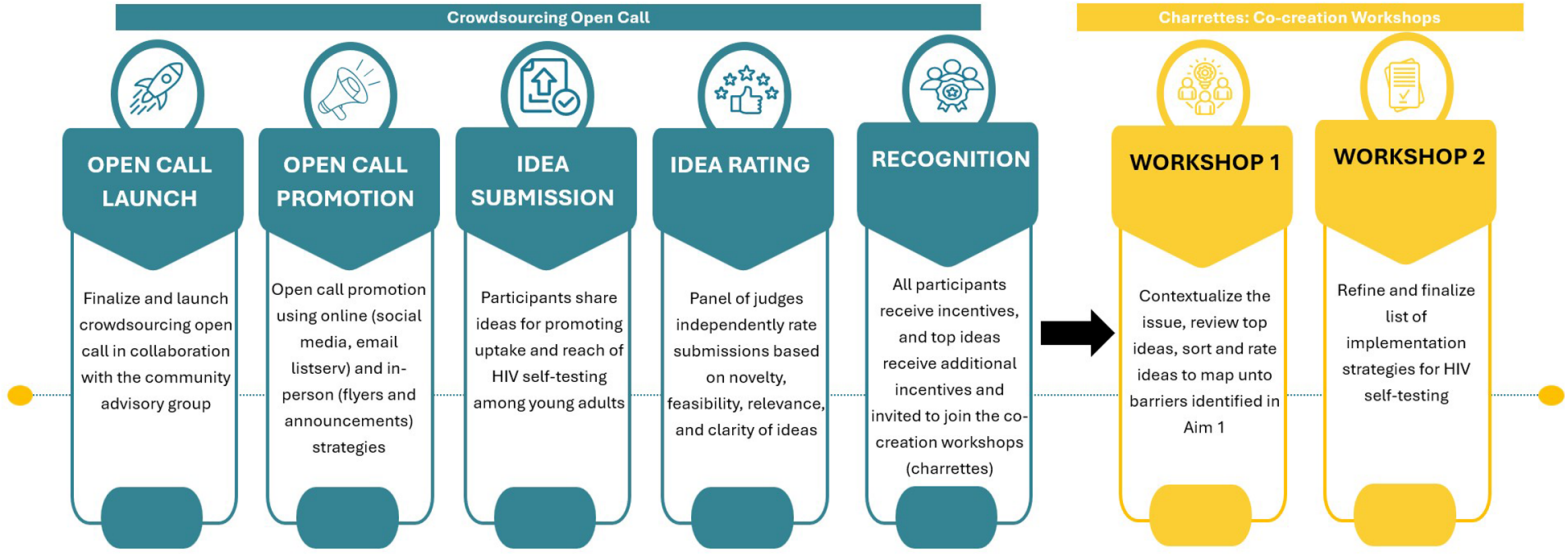
Overview of phase 2 of the study-crowdsourcing open call and charrettes.

Participants for the crowdsourcing open call will include a broad range of individuals, with a focus on ensuring that each group has at least one young adult aged 18–29 years residing in or attending school in North Carolina. This inclusive approach allows for a diverse range of contributions while ensuring that the perspectives of young adults are central to the development of HIVST implementation strategies (39). The open call will be announced on the social media platforms of our community organization partners and in person, using flyers to raise awareness. Eligible open-call entries will include submissions from individuals or groups of individuals (teams) that: (1) respond in English; (2) reside in North Carolina at the time of the open call; and (3) be willing and able to provide informed consent for the study (Figure 2).

##### Procedure - crowdsourcing open call [phase 2.1]

###### Role of CAG

Similar to our previous open calls (31, 32), we will establish an crowdsourcing open call in collaboration with the CAG. The CAG will be pivotal in every phase of the open call, including planning, implementing, and evaluating the crowdsourcing open call. During the planning phase, the CAG will collaborate with the research team to design the open call, ensuring it is young adults-friendly and engaging. Additionally, the CAG will assist with promoting the open call through their social media platforms and networks during the implementation phase. The CAG will participate in the evaluation phase of the judging panel to assess the submissions.

###### Open call process

The open call will be announced on the social media platforms of our community organization partners to raise awareness. The open call will solicit contributions from young adults in the form of images, videos, posters, and written concepts (minimum 250 words) on ways to promote HIVST. The open call will be promoted online through posts on the project website, popular social media platforms used by young adults, such as Instagram, Facebook, LinkedIn, TikTok, X, YouTube, and community organizations' websites. The project website will also serve as a channel for announcing prizes, deadlines, and other relevant information. In-person events will include community-based introductions, interactive feedback sessions, and community-driven events (decided by community partners). Decisions about types and amounts for appropriate incentives (e.g., tablets, cash) and participatory prizes (e.g., certificates) for participants will be agreed upon by members of the CAG to avoid coercion while encouraging young adult participation.

Submitted ideas will be evaluated to ensure that copyright issues are covered, and a question will be included to confirm that each submission is original material from the participants. Additionally, all participants will complete a waiver of use or ownership to release their ideas for implementation strategy development. The CAG will organize a panel of judges, including public health professionals, community members and partners, representatives from the CAG, researchers, and young adults. Submissions will be assessed using an adapted crowd idea quality assessment tool, which considers four dimensions: novelty, relevance, feasibility, and clarity of idea (40). The convened panel of judges will evaluate each anonymous idea entry using the crowd idea quality assessment tool (41). The CAG and the research team will convene and reach a consensus to select the final top ideas to be invited to the charrettes (Phase 2.2).

##### Participants and recruitment - charrettes: co-creation workshops [phase 2.2]

Charrettes are intensive participatory workshops that involve community members in co-learning to solve a specific community problem (42). Members of the CAG, the study team, members of the community-based organizations, and the young adults with the top ideas from the crowdsourcing open call will be invited to participate in a two-day charrette to iteratively finalize components of HIVST implementation strategies based on the contributed crowdsourced ideas. The charrettes will be scheduled based on the availability of the community partners, community advisory group members, and the young adults with the top ideas from the crowdsourcing. We will also provide hybrid formats (in-person and online engagement) to accommodate participants' preferences.

##### Procedure - charrettes: co-creation workshops [phase 2.2]

The charette will occur over two co-creation workshops. In preparation for the workshops, the research team will develop preliminary implementation strategies based on the top ideas generated from the crowdsourcing open call. Workshop 1 will commence with brief presentations that provide an overview of the project, barriers and facilitators to HIVST implementation identified in Aim 1, an overview of the top ideas from the crowdsourcing open call, and the goals of the charrettes. Additionally, this workshop will involve discussions to review the selected crowdsourced ideas and identify components that will be useful for promoting HIVST. Structured evaluations will be conducted to rate the components’ relevance and feasibility and to select the most important and feasible ones. Card sorting and rating will be used, an established technique wherein participants group items into categories based on similarity or difference and subsequently rank them according to salience and feasibility (43–46). Workshop 2 will involve refining and finalizing implementation strategies for HIVST. Prototyping techniques such as personas (understanding user needs through hypothetical archetypes of actual users) and scenarios-of-use (specific examples of how users, context, and intervention interact) (47) will be used to generate prototypes of the implementation strategies for HIVST. At the end of the second workshop, the research team will work with the participants to synthesize and finalize the list of implementation strategies for HIVST. Multiple implementation strategies may be identified during the workshops, and some may be more relevant for particular sub-groups. Therefore, the co-creation workshops will be instrumental in identifying and selecting the top strategies that may be bundled to increase HIVST among young adults. Because this is a pilot study, implementation strategies that are narrowly focused on one group may be used to support a future study. Moreover, we anticipate that multiple strategies to deliver kits securely and confidentially will be co-created during the workshops (i.e., distribution sites, preferred locations, delivery methods, packaging, etc.).

##### Analysis

Summary statistics will describe the sociodemographic factors of individuals who participated in the open call. We will cross-tabulate the responses by sociodemographic characteristics and explore differences using bivariate analyses (chi-square tests and *t*-tests). Data visualizations will be created using LASSO penalization, including network visualizations of polychoric and tetrachoric correlations among sociodemographic characteristics. Variables composing the network edges will be summarized according to common centrality metrics (e.g., strength, closeness, etc.). Subnetworks will be explored using key sociodemographic characteristics. We will analyze the qualitative data from crowdsourcing open call submissions and charrettes workshops using a framework approach for qualitative data analysis (37), as described in Phase 1. Codes will be developed stepwise using a comprehensive code structure created by the team to capture all data concepts and emergent themes. Concepts will then be grouped into categories and themes reflecting the findings generated and incorporated in finalizing the implementation strategies for HIVST.

##### Expected outcome

Phase 2 of the project will culminate in finalized co-created HIVST implementation strategies, which will be piloted in Phase 3 of the study.

#### Phase 3: implementation and evaluation - implement co-created HIVST implementation strategies among young adults and measure implementation outcomes (primary outcomes) and preliminary effectiveness outcomes (exploratory)

Phase 3 includes implementing the finalized implementation strategies from Phase 2 over three months in collaboration with participating community partners.

##### Participants and recruitment

Approximately 240 participants aged 18–29 will be recruited to participate in the co-created implementation strategies for HIVST. Our choice of sample size (*N* = 240) is based on feasibility, as documented by similar studies (48, 49). This sample size will provide reasonable estimates of key parameters, including the uptake of HIVST among young adults based on implementation strategies, and inform the sample size needed for future studies among this population. Although this study is not powered, we will collect data on effectiveness outcomes to inform future large-scale studies.

In collaboration with the two implementing community partners (RAIN, Inc. and RAO Community Health), we will recruit a diverse sample of young adults by using social media and other online platforms (including websites and social media accounts operated by local organizations), event- and venue-based activities, and participants' referrals at community centers serving young adults. Inclusion criteria include: 18–29 years old, report of previous sexual intercourse with another individual or injection drug use, resident of North Carolina for the next 3 months, and informed consent. Exclusion criteria include self-reporting living with HIV.

##### Procedure

Participants who consent to participate will be directed to a secure electronic REDCap platform to learn more about the strategy. Baseline data on participants' self-reported sociodemographic characteristics, HIV testing history, sexual behavior history, HIV risk behaviors, STI testing, STI treatment among those with infection, PrEP use, and condom use will be collected. No biological specimens will be collected. We will create a unique identifier to match participants at baseline and follow-up. Access to survey data will be restricted to relevant study team and stored on secure, password protected university issued computers. Linking identifying information (i.e., email) will be stored separately from survey data. After consenting and completing the baseline survey, participants will receive the content of the implementation strategy. The strategy will be a bundle that includes components of the finalized co-created HIVST implementation strategies. For example, this may include promotional messaging and a description of how to receive the HIVST based on the finalized strategy.

As part of the pilot implementation, we will use the RE-AIM framework to identify key outcomes related to the implementation and effectiveness of the co-created HIVST implementation strategies. We will collect data on *Reach, Effectiveness, Adoption, Implementation*, and *Maintenance*. We will collect and assess implementation strategy reach through basic de-identified sociodemographic data (i.e., participants’ age, race/population group). *Reach* will be measured as the total number and summary characteristics of individuals recruited to participate in the study. *Effectiveness* will be measured as the impact on clinical outcomes testing, comparing the pre-post change in the proportion of HIV testing among study participants. The data will be obtained based on self-reported information on HIV testing among study participants comparing baseline and 3-month follow-up (preliminary effectiveness parameter). *Adoption* will be measured as the proportion and representativeness of participants who receive an HIVST at 3-month follow-up. *Implementation* will be measured using brief, validated psychometric instruments that are considered robust predictors of implementation success and will be administered at 3-month follow-up: Acceptability of Intervention Measure (AIM), Intervention Appropriateness Measure (IAM), and Feasibility of Intervention Measure (FIM) with four items scored on a 5-point scale (50). In addition, we will obtain implementation cost data to estimate the test's cost-per-unit range. Maintenance will be measured by the utilization of implementation strategies at study sites post-intervention and the sustained intentions of study participants for HIV self-testing post-intervention. We will conduct exit interviews at the end of the implementation period (within one month of implementation completion). For this, we will recruit approximately 20 participants to explore their experiences with the implementation strategies, barriers, facilitators, and opportunities with the uptake of the strategy, as well as recommendations for improving HIVST implementation. Participants will be selected for interviews using purposive sampling based on the level of engagement in the study (high or low engagement relative to other participants). High engagement would be individuals who respond to both baseline and follow-up surveys, and low engagement would be individuals who respond to only the baseline surveys. We will also interview representatives from the implementing community organizations (∼6) to understand their experiences with the implementation, including barriers, facilitators, and opportunities they encountered and perspectives of sustaining HIV self-testing post-study implementation. For example, questions will ask HIVST recipients and implementation partners about their intentions to use HIVST kits and the piloted implementation strategies over the next 12 months.

Participants completing the surveys will be offered a $30 gift card for each survey they complete (baseline and 3-month follow-up). Additionally, participants who take part in interviews will receive a $50 gift card.

##### Analysis

Sociodemographic characteristics and data on implementation outcomes - reach, effectiveness, adoption, and implementation - will be analyzed descriptively, including means, standard deviations, and ranges for continuous data and frequencies and percentages for nominal data. Pre-post differences in the psychometric scores will be analyzed upon adjusting for sociodemographic characteristics, and adjusted differences with 95% confidence intervals will be reported. Network visualizations with LASSO penalizations will also be performed to explore associations within and between sociodemographic characteristics and psychometric scores. Strong adjusted associations between sociodemographic characteristics and differences in psychometric scores will be identified and reported. Generalized linear regressions were used to compare the differences between the two-time points (baseline and 3-month follow-up) in the proportion of young adults tested for HIV. Although we will attempt to retain as high a fraction of participants as possible, we acknowledge that some attrition is likely, leading to missing outcome values (48). The generalized linear regression proposed for the primary analyses incorporates an assumption of missing data at random (MAR), meaning that the likelihood of a value being missing depends on observable characteristics (e.g., sex or age). We will use sensitivity analyses to assess the impact of different assumptions about the missing data mechanism and outliers. We will further determine the robustness of the results based on these different assumptions. We will consider using multiple imputations of missing data as an alternative to sensitivity analysis. Qualitative Data: Data collected from in-depth interviews will be transcribed verbatim and entered in NVivo, version 14, for coding and subsequent qualitative data analysis. We will analyze the data from the interviews by using a framework approach for qualitative data analysis (37), as described in Phase 1.

##### Expected outcome

In Phase 3 of the study, we will obtain implementation outcomes data describing the reach, adoption, and implementation of co-created HIVST implementation strategies. Although not powered, we will also explore preliminary measures of the effectiveness of the co-created strategies, maintenance indicators, and a preliminary implementation cost analysis.

###### Data management

All data will be stored on a password-protected computer and password-encrypted servers. Only members of the research team will have access to the raw data. Consent forms will be stored separately from participant data, and each participant will be assigned a unique identifier code. Data will be stored for a maximum of 5 years before being securely destroyed.

## Discussion

This protocol describes the CATEST study, which seeks to utilize participatory approaches to co-create implementation strategies to promote the uptake of HIVST among young adults in Mecklenburg County, NC. The CATEST study addresses the need to promote HIV prevention among young adults in Mecklenburg County, *a priori*ty EHE area (23). Innovative approaches to promote HIV testing and to engage young adults in the uptake of HIV prevention services are urgently needed to reduce new HIV infections (51, 52). Yet, few interventions have engaged young adults in the development and implementation process. Engaging a diverse group of young adults in developing HIVST implementation strategies can lead to developing more inclusive strategies that are better utilized, appropriate, and more acceptable to young adults (53). The CATEST study proposes adopting collaborative approach, where young adults are recognized as change agents to improve their health (54–56).

Following the principles of CBPR (17, 18, 57), this project focuses on creating partnerships with local community organizations to support the implementation of the study activities and on engaging young adults in the co-creation activities for the study. We will forge partnerships to garner the wisdom of young adults, local communities, organizations, and health systems to develop HIVST implementation strategies to augment current HIV prevention efforts in Mecklenburg County. The project has the potential to contribute to the County's EHE efforts by engaging young adults within local communities in developing community-centered strategies that resonate with and elevate the voices of local communities. In addition, this pilot study will provide important preliminary findings on the feasibility, acceptability, and preliminary effectiveness of the co-created HIVST implementation strategies, informing a full-scale trial and the growing literature on leveraging participatory approaches to co-create implementation strategies to enhance young adults' awareness and uptake of HIV interventions.

This study has implications for young adults; we foster a strength-based approach to intervention development and implementation by actively engaging young adults and key community partners. The collaborative engagement allows for the meaningful involvement of young adults and key community partners, to foster trust and leverage their strengths and capabilities (58). Such community-engaged and participatory approaches have shown promise in enhancing the relevance and acceptability of an intervention and contributing to its long-term sustainable use (59–61). The study outcomes may help to guide intervention development and implementation practices among young adult-serving community-based organizations, fostering demand for and enhancing uptake of HIV prevention and other services among young adults.

### Limitations

Findings should be interpreted in light of possible study limitations. The pilot study participants will be enrolled in two community-based organizations that provide HIV services, which means that results may not be generalized to other young adults in the region. However, there are limited instances of young adults co-creating implementation strategies for HIVST, so this information may contribute new knowledge regarding the feasibility and implementation of such strategies. Lessons learned from this study can inform future implementation in other community organizations. Additionally, some of the behavioral measures, such as sexual behavior history, will be self-reported data that are prone to social desirability bias (48). Based on experience with previous studies (59, 48) to minimize the risk of social desirability bias, our protocol includes establishing trust through the informed consent process and using an online survey data collection process that ensures participants' privacy and anonymity as they complete the questionnaires. In addition, regarding the co-creation phase of the project, there is a potential risk of selection bias. There is a tendency for young people who are already interested in the topic to participate in the crowdsourcing open call. To mitigate this challenge, we would utilize several recruitment strategies in collaboration with community partners to reach and recruit a diverse group of participants. In addition, in the case of low engagement among the focus audience, we will utilize other co-creation methods such as implementation mapping or human-centered design approaches that allow for co-production and active participation of end-users in the intervention development and implementation processes. Lastly, this is not a longitudinal study, which presents challenges with measuring maintenance within the pilot study timeframe.

### Conclusions

The CATEST project aims to develop novel, participatory, and community-driven implementation strategies to promote HIVST among young adults in Mecklenburg County, NC. Our study will provide valuable insight into the feasibility, reach, and adoption of co-created implementation strategies for HIVST among young adults and explore preliminary measures of effectiveness and maintenance to inform future larger-scale studies. CATEST has the potential to serve as a model and roadmap for widespread young adult engagement and the implementation of HIVST using equitable and accessible strategies. Additionally, this study can offer exemplars of participatory approaches for co-creating implementation strategies.

#### Study status

At the time of this publication, the analysis of the interviews from Phase 1 of the study is ongoing. Recruitment for Phase 2 of the study will begin in November 2024 and will end in February 2025. Phase 3 of the study is expected to begin in March 2025 and be completed by the end of June 2025.

#### Dissemination plans

Findings from this study will be communicated to key communities of interest through dissemination meetings using language-appropriate communication materials. Results will be presented at a national academic conference and published in open-access peer-reviewed journals focused on HIV, implementation science, behavioral science, public health, and the social sciences. To reach people beyond the academic community, we will also utilize social media platforms to disseminate key study findings and explain study goals and strategies. The dataset generated from this protocol will be available after the primary analysis is completed and published and can be accessed by contacting the corresponding author.

## Ethics statement

This protocol described herein was approved by the Wake Forest University School of Medicine Institutional Ethical Review Board [IRB# IRB00099305], and ethical reliance was obtained for the University of North Carolina at Charlotte. All participants in this study will undergo consenting procedures.
